# Typical food portion sizes consumed by Australian adults: results from the 2011–12 Australian National Nutrition and Physical Activity Survey

**DOI:** 10.1038/srep19596

**Published:** 2016-01-20

**Authors:** Miaobing Zheng, Jason H Y Wu, Jimmy Chun Yu Louie, Victoria M Flood, Tim Gill, Beth Thomas, Xenia Cleanthous, Bruce Neal, Anna Rangan

**Affiliations:** 1 School of Molecular Bioscience, Charles Perkins Centre, The University of Sydney, Sydney NSW Australia; 2 The George Institute for Global Health, Sydney Medical School, The University of Sydney, Camperdown NSW Australia; 3 Faculty of Health Sciences, The University of Sydney, Sydney NSW Australia; 4 St Vincent’s Hospital, Darlinghurst NSW Australia; 5 Boden Institute of Obesity, Nutrition, Exercise and Eating Disorders, The University of Sydney, Sydney NSW Australia; 6 National Heart Foundation of Australia, Melbourne VIC Australia

## Abstract

Considerable evidence has associated increasing portion sizes with elevated obesity prevalence. This study examines typical portion sizes of commonly consumed core and discretionary foods in Australian adults, and compares these data with the Australian Dietary Guidelines standard serves. Typical portion sizes are defined as the median amount of foods consumed per eating occasion. Sex- and age-specific median portion sizes of adults aged 19 years and over (n = 9341) were analysed using one day 24 hour recall data from the 2011–12 National Nutrition and Physical Activity Survey. A total of 152 food categories were examined. There were significant sex and age differences in typical portion sizes among a large proportion of food categories studied. Typical portion sizes of breads and cereals, meat and chicken cuts, and starchy vegetables were 30–160% larger than the standard serves, whereas, the portion sizes of dairy products, some fruits, and non-starchy vegetables were 30–90% smaller. Typical portion sizes for discretionary foods such as cakes, ice-cream, sausages, hamburgers, pizza, and alcoholic drinks exceeded the standard serves by 40–400%. The findings of the present study are particularly relevant for establishing Australian-specific reference portions for dietary assessment tools, refinement of nutrition labelling and public health policies.

In light of the far-reaching health and social implications of overweight and obesity, obesity prevention and improving the health status of populations have become critical concerns worldwide^1^. In 2011–12, approximately 63% of Australian adults were overweight, of which 28% were obese^2^. Poor diet along with overweight and obesity have been recognised as the leading risk factors of disease burden in Australia^3^. The fundamental cause of overweight and obesity is energy imbalance with energy intake exceeding energy expenditure attributable to complex interactions of genetics, environmental, dietary and behavioural factors^4^. Increasing portion sizes may contribute to excess energy intake, and development of obesity and chronic diseases^5^,^6^,^7^.

The Australian Dietary Guidelines (ADG) encourage people to focus on appropriate portion sizes of ‘core’ foods and avoid ‘discretionary’ foods for optimal nutrition and health^8^. Foods from the core food groups including bread and cereals, meat and alternatives, milk and alternatives, fruit, and vegetables form the basis of a healthy diet^8^. Discretionary foods are defined as energy-dense and nutrient-poor foods that are unnecessary in a healthy diet^8^. In the ADG, standard serves and the recommended number of serves guide individuals to achieve healthy food and nutrient intakes. However, the 2011–12 National Nutrition and Physical Activity Survey (NNPAS) indicates a disconnect between dietary guidelines and what people actually consume. Only 54% and 7% of Australians met the recommended intake of fruit and vegetables, respectively^2^. In contrast, discretionary foods contributed 35% of total energy intake of Australians^2^. A lack of awareness among the public regarding appropriate portion sizes and how portion sizes relate to the standard serves and recommended number of serves in the ADG may be a contributing factor^9^.

Typical portion sizes are defined as the median amounts of foods consumed per eating occasion^8^. However, results of national surveys usually report food intake on a per day basis, and do not specify portion sizes of foods consumed per eating occasion^2^. Most studies on typical portion sizes are limited to specific foods such as those high in energy and poor in nutrients^10^,^11^, or those contributing most to energy intake^12^. The aim of this study was to update our previous portion size analysis which used the 1995 Australian National Nutrition Survey^13^ and to examine the typical portion sizes of a wide range of commonly consumed core and discretionary foods among Australian adults using the 2011–12 NNPAS, and to evaluate the differences between typical portion sizes consumed and the ADG standard serves.

## Results

### Portion sizes of commonly consumed food categories

The current analyses included a total of 152 food categories. Of these, 97 were core foods and 55 were discretionary foods. Median portion sizes of selected commonly consumed core and discretionary food categories by sex and age are presented in Tables 1 and 2, respectively. A complete list of all food categories analysed are presented in the online supplementary material (Supplementary Tables 1, 2, 3 and 4 online).

Regardless of core or discretionary food categories, there were significant sex differences in typical portion sizes. The typical portion sizes for males were significantly larger than for females in 76% (115 out of 152) of food categories studied (P < 0.05) (Supplementary Tables 1, 2 online). Typical portion sizes of some food categories such as breakfast cereals, cooked pasta, noodles or rice, chicken, some vegetables, sweet biscuits, sausages, butter, sports and energy drinks, beer, and pizza were more than 30% larger for males compared to portions consumed by females. Significant age differences in typical portion sizes were observed for 68% (103 out of 152) of food categories studied including most breads and cereals, meat and alternatives, milk and yoghurt, most fruits, some vegetables, sweet biscuits, cakes, sausages, sugar-sweetened beverages, and alcoholic drinks (Supplementary Tables 1, 2 online). Typical portion sizes decreased with increasing age, where adults aged 71 years and over had the smallest portion sizes compared to the younger age groups. Typical portion sizes of food categories that did not have significant age differences were cooked oats, pasta, cheese, custard, mandarins, cooked carrot, cucumber, boiled potatoes, margarine, bacon, ham, savoury sauces and condiments such as gravies, pasta sauce and salad dressing. The portion sizes also varied by the use of that given food or beverage, for example, the amount of milk used in beverage was smaller than milk used on cereal (Tables 1 and 2).

Food categories with a wide portion size range included cooked oats, pasta, noodles, rice, grapes, cooked beans and legumes, baked potatoes, pizza, burgers and other mixed dishes including rice, pasta, meat or vegetables. Portion size variations were particularly large for beverages such as water, milk on cereal, flavoured milk, fruit juices, sugar-sweetened beverages, and alcoholic drinks (Tables 1 and 2).

### Comparison with the ADG standard serves

There were obvious differences between the NNPAS typical portion sizes and the ADG standard serves (Tables 3 and 4). The portion sizes for food categories under the bread and cereal group were generally larger than the ADG standard serves (e.g. 40 g of bread and 30 g of breakfast cereals). Greater differences were observed in males than in females. Bread portion sizes for males and females exceeded the ADG standard serve by approximately 60% and 40%, respectively. Portion sizes for all ready-to-eat breakfast cereals for males and females were 70% and 18% larger than the standard serve. Breakfast cereals such as wheat-flake biscuits, muesli, and mixed grain flakes contributed the largest differences to the standard serve. Likewise, the portion sizes for cooked cereals including oats, pasta, noodles and rice were significantly larger than the standard serve.

Within the meat and alternatives, meat portions including beef, lamb, chicken and pork were about 90–160% greater than the standard serve. Portion sizes for fish, seafood, and nuts were similar to their standard serves (within ±25%), whereas the portion size for eggs was half of its standard serve. For dairy products including milk, yoghurt, cheese and milk alternatives, the portion sizes were generally smaller than the standard serves, except for flavoured milk, which had a 40–80% greater portion size. With respect to fruits, median portion sizes of apples, pears and oranges were similar to the 150 g standard serve. In contrast, small sized fruits such as mandarin, strawberries and grapes had portion sizes 40~60% smaller than the standard serve. Vegetables such as beetroot, carrot, lettuce, cucumber, tomato were consistently smaller than the 75 g standard serve in both males and females, except for cooked legumes, baked beans, starchy vegetables (i.e. corn and potatoes), and cooked mixed non-leafy vegetables.

Typical portion sizes of discretionary foods such as cakes, buns, muffins, doughnuts, sweet pastries, sausages, hamburgers, pizza, savoury pastries, potato fries, and alcoholic drinks exceeded the 600 kJ standard serves by 40–400%. In contrast, the portion sizes for sweet biscuits, ham, chocolate, potato crisps, and sugar-sweetened beverages were similar to 600 kJ standard serve.

## Discussion

The current analyses examined the typical portion sizes of commonly consumed core and discretionary foods among Australian adults, and assessed how these compared to the ADG standard serves. There were significant sex and age differences in typical portion sizes among a large proportion of food categories studied, regardless of their classification as core or discretionary foods. Comparison of typical portion sizes with ADG standard serves revealed consistent trends among core food categories with breads and cereals, meat and chicken cuts, and starchy vegetables being consumed in larger amounts than their standard serves. In contrast, the portion sizes of milk, yoghurt, cheese, small sized fruits, and non-starchy vegetables were generally smaller than their standard serves. Portion sizes of discretionary foods such as cakes, potato fries, hamburgers, pizza, sausage, beer, and wine exceeded the 600 kJ standard serve by approximately 40–400%.

The sex- and age-specific typical food portion sizes found in the present study are consistent with previous portion size literature^13^,^14^,^15^,^16^. The sex differences in portion sizes may be a consequence of greater energy requirement of males relative to females. Furthermore, it has been suggested that females’ stronger beliefs in healthy eating and greater involvement in dieting and weight control may partly contribute to the sex differences in eating habits^17^,^18^, and potential better portion size control of females compared with males^19^. Not surprisingly, the 2011–12 AHS found that dieting behaviours were more prevalent in females than in males^2^. The smaller portion sizes among elderly people may be partially explained by the decreased energy requirement attributable to both lower metabolic rate and physical activity level.

ADG standard serves together with the number of serves per day, help people to quantify the total daily amounts of food required for nutrient and energy requirements^8^. However, evidence has shown standard serves are generally not well understood by the public^20^. A Canadian study found participants (n = 145) overestimated standard serves (e.g. grains, fruit and vegetables) and underestimated the number of serves they consumed^20^. It is noteworthy that public health messages may overlook the fact that people have different interpretations of a standard serve and more education in this area is needed. As indicated by current results, core foods with typical portion sizes smaller than standard serves (e.g. milk, yoghurt, cheese, non-starchy vegetables, and some fruits) will need to be consumed more frequently to ensure dietary guidelines are met. The findings that typical core food portion sizes of bread, cereals, meat and chicken cuts being larger than the standard serves were similar to the findings of a small Australian pilot study, which asked participants to serve themselves the typical amount of foods that they normally consume at one eating occasion^21^. This study also found that self-selected portion sizes of rice, pasta, breakfast cereal, and meat were larger than the ADG standard serves, except for milk, which was smaller^21^.

Portion sizes of discretionary foods are particularly relevant to the current climate of excess energy intake and overweight/obesity. The ADG recommend adults limit their consumption of discretionary foods to 0–3 serves depending on their energy requirement^8^. Typical portion sizes of some discretionary food categories in this study were approximately 40–400% larger than the ADG standard serve of 600 kJ, highlighting the importance of targeting these food categories to reduce excess energy intake. Typical portion sizes of pizza and hamburgers were 2–4 times higher than a standard serve, while cakes and muffins, and pastries were 1.5 times higher. In contrast, typical portion sizes of soft drinks, fruit drinks, sweet biscuits, chocolate, and potato crisps were similar to a standard serve of discretionary food. Foods typically consumed in small portion sizes (e.g. <20 g) such as sugar, honey, jam and butter contributed significantly lower energy compared with a standard serve.

The accuracy of portion size estimation is an important limitation to our study. Studies have shown portion size estimation is difficult for nearly all people, regardless of age, sex, body weight or socio-economic status^22^. Foods that are particularly difficult to estimate include amorphous foods that take the shape of the container they are in^23^,^24^, foods in small quantities^25^, and foods with multiple components such as mixed dishes^26^. Indeed, large variations were observed in typical portion sizes of cooked cereals, beverage, and mixed dishes in this study. Moreover, portion size may be deliberately misreported. People may report smaller portion sizes of discretionary foods or larger portions of fruits and vegetables for social desirability reasons but this could not be measured in our study^27^. The current study has several strengths. The current analyses were based on a large sample of national representative Australian population. The utilisation of measurement aids such as real-sized food and container images in 24 hour recall has been demonstrated to improve the accuracy of the portion size estimates^28^, but there is also potential for these measures to influence the recall of portion size. For example, the median portion sizes of some food categories in the current analyses were similar to the portion size depicted in the food model booklet such as meat cuts, fruits, and beverages.

In conclusion, this study examined the most up-to-date typical portion sizes of a comprehensive list of commonly consumed core and discretionary food categories in the Australian population. These findings can be used in clinical practice to assist health educators in nutrition counselling; in nutrition research such as the development of dietary assessment tools and in public health nutrition to monitor food consumption trends, design public health campaigns, refine nutrition labelling and support public health policies and guidelines. Future dietary interventions and public health campaigns should focus on education regarding portion size. It is important to clarify that a general message to decrease portion size of all foods is not appropriate. Future guidance should encourage people to increase the portion of low energy, nutrient rich core foods such as fruit and vegetables, and decrease portions for energy dense and nutrient poor discretionary foods.

## Methods

### Study design and participants

This study used data from adults aged 19 years and over (*n* = 9341) who participated in the 2011–12 NNPAS. The 2011–12 NNPAS, focused on the collection of dietary intake and physical activity information, and was a component of the 2011–13 Australian Health Survey (AHS)^29^. The NNPAS was conducted throughout Australia from May 2011 to June 2012 within approximately 9,500 private dwellings (77% of participating dwellings) across Australia. A stratified multistage area sampling was used for sample selection, and to ensure the selected sample was representative of the Australian population. One face-to-face 24 hour recall, which was collected using the five-phase automated multiple-pass method, was used to analyse the typical portion size data^29^. Participants were asked to report all foods consumed on the day prior to the interview, from midnight to midnight. The dietary intake data were coded to an Australian Food, Supplement and Nutrient Database (AUSNUT) 8-digit food code and categorised into food classification groups based on the food and measures database developed by Food Standard Australia New Zealand (FASNZ). Detailed study design and operation have been reported on the Australian Bureau of Statistics (ABS) website^29^.

### Determination of portion size

The portion size of a food was defined as the amount of food that an individual consumed at one eating occasion. A food model booklet containing the actual size photographs and drawings of different-sized Australian sourced beverage and food containers, shapes and mounds, ruler, rings, a grid, a wedge, meat and chicken cuts, and chocolate bar sizes were provided to assist respondents with portion size estimation^29^. Respondents were asked for the time they began eating or drinking each food as well as what the respondent would call each eating occasion^29^. If an individual consumed a food item multiple occasions a day, the average portion size for those multiple occasions was calculated and treated as a single record for that individual.

### Classification of food categories

The classification of food categories was based on grouping similar foods together using the AUSNUT 8-digit food codes consistent with our previous analysis^13^. Food categories were classified further into core and discretionary foods according to the ADG^8^ and the ABS discretionary food list^30^, respectively. The bread and cereal group consisted of different types of breads, breakfast cereals, cooked oats, rice, pasta and noodles. The meat and alternative group included cooked beef cuts, lamb cuts, pork cuts, chicken, fish, other seafood, eggs, nuts, and meat alternatives. The milk and alternatives group included milk, yoghurt, and cheese of varying fat types, custard, flavoured milk, and milk substitutes. Milk was further classified based on its use: ‘in beverage’ or ‘on cereal’, according to the food combination code: ‘beverage with additions’ and ‘cereals with additions’. The fruit group was comprised of commonly consumed pome, berry, citrus, tropical, and dried fruits (such as apples, pears, strawberries, bananas, and raisins) and pure fruit juices. The vegetable group included cooked green and orange vegetables, raw salad vegetables, cooked legumes/beans, and starchy vegetables. The portion sizes of unsaturated spreads and oils such as margarine and olive oil; and fluids including water, tea and coffee were also assessed. Discretionary foods included pizza, hamburgers, potato fries, processed meats, biscuits and cakes, confectionary and snacks, sugar-sweetened beverages, sauces and condiments, sugar and spreads, and alcoholic drinks. Only foods and beverages reported as a single item were captured in the above food categories. Mixed dishes were coded as a single item if insufficient detail was given to code as multiple items such as soups, savoury rice dishes, savoury pasta/noodle dishes, vegetable dishes and meat dishes.

### Comparison with ADG standard serves

In the ADG, the standard serves of core foods were determined according to the average weight of current household measures or of usual consumption units, taking into account the nutritional contribution of the food item in total diets^8^. The standard serves for discretionary foods were established on the basis of 600 kJ cut-off. The NNPAS median portion size of individual food categories by sex was compared to the ADG standard serves. The percentage difference between NNPAS median portion size and ADG standard serve was calculated as (median portion size–standard serve)/standard serve ×100. The standard serve for beverages given in volume (mL) were converted to grams based on the density measures provided by the AUSNUT food measures file^31^. For example, the recommended standard serve of regular milk (250 mL) is equivalent to 258 g based on the density of 1.03 g/mL^31^.

### Statistical analysis

Data were analysed according to sex and the following age groups: 19 to 30 years (*n* = 1592), 31 to 50 years (*n* = 3565), 51 to 70 years (*n* = 2906), and 71 years and over (*n* = 1278). Median portion sizes, 25^th^ and 75^th^ percentiles (grams) were determined for food categories in all sex and age subgroups with more than ten consumers. Differences in median portion sizes by sex were tested using Mann-Whitney tests. Kruskal-Wallis tests were conducted to compare the median portion sizes across the four age groups. Median energy per portion (kJ) of discretionary foods was determined for comparison with the 600 kJ standard serve. One sample Wilcoxon Signed Rank tests were performed to test the differences between median portion sizes and the ADG standard serves. Percentage differences within 25% were considered as similar. Personal weighting factors were applied to the dataset to ensure that the survey estimates conform to the population estimates by sex, age, area of usual residence and seasonal effects^29^. All statistical analyses were performed using SPSS 20.0 (SPSS Inc, Chicago, IL, USA) with statistical significance set as P < 0.05 (two-sided).

## Additional Information

**How to cite this article**: Zheng, M. *et al.* Typical food portion sizes consumed by Australian adults: results from the 2011–12 Australian National Nutrition and Physical Activity Survey. *Sci. Rep.* **6**, 19596; doi: 10.1038/srep19596 (2016).

## Supplementary Material

Supplementary Tables

## Figures and Tables

**Table 1:** Median portion sizes and interquartile range (grams) of commonly consumed core foods among consumers by age and sex (2011–12 NNPAS)^1^.

	19–30 years				31–50 years				51–70 years				71 + years				Overall M:F
	Male		Female		Male		Female		Male		Female		Male		Female		
	Median	IQR	Median	IQR	Median	IQR	Median	IQR	Median	IQR	Median	IQR	Median	IQR	Median	IQR	
Breads and cereals																	
Bread rolls, white	69	69–108	69	35–69	69	69–118	69	64–69	69	69–76	69	52–69	69	52–74	69	34–69	1.00
Bread, white	68	54–97	60	54–66	64	54–82	54	38–64	64	54–75	54	35–68	60	45–70	49	33–64	1.19
Bread, wholemeal	66	59–85	60	56–66	65	56–91	56	42–66	66	56–82	60	38–68	59	44–76	56	33–66	1.00
Breakfast cereal, all^2^	68	47–102	45	34–65	51	34–83	39	30–66	51	37–78	34	25–59	35	25–52	31	17–39	
Oats, cooked	312	201–404	198	104–315	260	195–484	199	104–260	218	130–468	202	156–312	202	156–333	202	130–312	1.08
Rice Bubbles/Corn Flakes	35	32–53	33	17–39	35	22–50	35	30–42	35	20–39	33	23–35	35	23–35	26	12–35	1.06
Rice, cooked	201	134–327	145	92–201	193	134–286	134	84–217	201	137–288	134	81–217	138	95–234	125	84–190	1.47
Wheat-flake biscuits	70	51–100	34	34–51	51	34–68	34	34–42	51	34–68	34	20–40	34	24–43	26	17–34	1.50
Meat and alternatives																	
Beef steak, cooked	186	150–205	156	126–186	171	147–200	150	106–171	169	144–198	147	100–184	133	75–184	150	104–179	1.13
Chicken cuts, cooked	174	104–246	113	93–186	176	100–238	112	75–186	166	105–226	113	76–186	113	87–188	112	76–186	1.51
Eggs, whole	51	42–98	51	44–84	53	47–88	47	42–84	51	44–88	49	44–80	69	42–94	47	42–52	1.04
Fish, cooked	121	81–161	107	91–128	110	61–145	101	66–110	119	91–201	110	86–135	124	90–210	110	101–122	1.03
Lamb, cuts, cooked	150	86–240	143	104–156	123	75–156	104	71–140	150	104–187	104	70–150	127	80–156	100	70–150	1.22
Nuts	31	22–44	28	15–36	28	15–36	28	13–36	30	21–41	27	12–36	27	13–36	14	9–28	1.04
Milk and alternatives																	
Cheese, cheddar, full fat	25	25–48	25	21–30	28	21–42	25	21–32	25	22–42	25	17–28	25	21–41	25	21–31	1.00
Milk, in beverage, full fat	41	31–134	41	31–103	31	31–53	31	31–72	31	31–62	31	31–46	31	31–46	31	31–41	1.00
Milk, on cereal, full fat	258	155–337	129	82–258	206	103–258	129	82–206	154	82–206	129	72–155	129	72–206	126	61–206	1.50
Yoghurt, flavoured, full fat	175	170–200	134	92–170	123	92–200	150	91–202	149	62–242	100	61–175	81	55–123	92	62–166	1.25
Fruit																	
Apples	164	164–188	164	151–173	164	158–180	164	143–164	164	153–182	164	139–164	139	139–164	139	77–164	1.00
Bananas	111	98–111	98	74–111	98	98–111	98	98–98	98	98–111	98	74–98	98	74–111	98	74–98	1.00
Fruit juices	315	263–473	273	210–368	305	210–378	263	158–353	263	210–368	210	95–305	210	147–305	210	106–301	1.04
Grapes	126	79–194	92	44–170	75	50–170	95	40–150	94	44–170	60	37–100	60	41–170	40	22–60	1.33
Mandarins	75	75–150	75	75–75	75	75–112	75	75–113	75	75–150	75	75–113	75	75–132	75	75–75	1.00
Pears	181	153–218	181	181–218	181	176–206	181	149–218	181	176–181	181	171–181	181	171–211	176	145–181	1.00
Vegetables																	
Broccoli, cooked	58	29–62	38	20–58	58	40–116	58	37–98	58	34–98	44	22–98	38	23–52	38	19–47	1.41
Carrot, cooked	53	18–106	37	25–71	60	30–114	49	25–80	77	34–116	39	20–78	45	30–77	53	30–78	1.54
Cucumber, raw	26	17–32	26	17–39	26	17–41	17	17–29	26	17–39	21	17–39	17	17–20	17	17–32	1.04
Green peas	38	27–80	38	26–80	50	26–118	38	12–64	51	32–81	38	13–80	38	13–73	38	13–80	1.05
Mixed vegetables	126	71–193	127	71–171	143	96–185	114	71–171	143	71–193	134	58–188	143	71–182	114	59–181	1.25
Potatoes, boiled	175	99–257	122	40–172	172	122–250	122	83–203	192	102–234	120	73–203	165	104–234	104	83–203	1.54
Salad, leafy	78	59–146	78	50–131	86	50–133	65	49–109	73	50–115	62	40–112	63	30–100	62	37–115	1.20
Tomato, raw	38	33–38	29	29–38	38	29–58	29	29–38	29	29–58	29	29–38	29	29–29	29	29–29	1.21
Fats																	
Margarine	10	5–14	5	5–10	7	5–12	5	5–10	7	5–10	5	5–10	6	5–10	5	5–10	1.40
Olive oil	18	13–18	18	17–18	18	9–18	18	5–18	18	9–18	10	5–18	9	9–18	9	6–18	1.00
Beverages																	
Coffee, made up	250	200–290	220	200–281	233	200–293	220	200–281	225	200–275	220	200–250	200	187–250	200	150–250	1.02
Tea, made up	250	200–330	239	200–330	233	200–330	220	200–293	225	200–300	225	200–263	215	200–250	213	175–250	1.00
Mixed dishes																	
Savoury pasta/noodles dishes^3^	399	230–572	312	208–450	395	208–520	327	166–468	338	208–468	312	132–421	349	105–421	333	192–508	1.22
Savoury rice dishes^4^	281	134–385	219	84–335	235	168–335	201	101–312	270	218–385	168	84–299	168	22–234	132	130–168	1.22
Soup, made up	420	207–560	357	275–515	420	306–674	309	206–515	502	303–695	357	272–592	303	206–463	309	203–407	1.08

^1^All data were weighted to represent population estimates. IQR: Interquartile range; M: F Male to female ratio; sample sizes varies within age/sex group as median intake were based on per consumer. Significance of sex- and age- differences in typical portion sizes was tested by Mann-Whitney test and Kruskal-Wallis test respectively. All food categories had significant sex- and age-differences except for cooked oats, mandarins, cooked carrot, boiled potatoes, full fat milk in beverage, olive oil, and savoury rice dishes.

^2^Breakfast cereal, all includes all ready-to-eat breakfast cereals such as bran, rice bubbles/corn flakes, wheat-flake biscuits, muesli, and mixed grain flakes.

^3^Savoury pasta/noodle dishes include all past and noodle sauce dishes, stir-fried noodle with meat or vegetables, pasta or noodle salad.

^4^Savoury rice dishes include paella, fried rice, risotto with egg, meat, or vegetables.

**Table 2:** Median portion sizes and interquartile range (grams) of commonly consumed discretionary foods among consumers by age and sex (2011–12 NNPAS)^1^.

	19–30 years				31–50 years				51–70 years				71 + years				Overall M:F
	Male		Female		Male		Female		Male		Female		Male		Female		
	Median	IQR	Median	IQR	Median	IQR	Median	IQR	Median	IQR	Median	IQR	Median	IQR	Median	IQR	
Foods																	
Cakes, buns, muffins	**110**	67–143	**114**	63–163	**110**	72–163	**96**	56–142	**95**	61–160	**81**	44–132	**81**	48–140	**76**	44–131	1.16
Savoury biscuits	**20**	11–42	**19**	12–30	**20**	12–38	**19**	12–28	**20**	12–29	**15**	10–28	**14**	11–25	**13**	7–19	1.18
Sweet biscuits	**37**	18–51	**31**	17–39	**29**	16–38	**20**	16–34	**25**	16–37	**18**	14–32	**22**	16–33	**18**	12–28	1.47
Ham	**17**	17–34	**17**	17–34	**18**	17–34	**17**	17–25	**17**	17–34	**17**	17–25	**17**	17–24	**17**	17–28	1.00
Sausages and frankfurts	**178**	89–202	**152**	89–178	**152**	86–202	**101**	89–178	**114**	89–178	**94**	82–178	**142**	89–178	**97**	76–135	1.50
Butter and dairy blends	**10**	5–12	**8**	5–10	**7**	5–10	**5**	5–10	**7**	5–10	**5**	5–10	**7**	5–10	**5**	5–10	1.40
Ice-cream	**102**	78–175	**80**	69–110	**83**	75–138	**75**	25–200	**83**	70–132	**78**	66–100	**83**	39–138	**69**	46–101	1.06
Honey	**29**	14–29	**14**	7–28	**14**	7–29	**11**	7–25	**14**	7–23	**7**	7–11	**11**	7–29	**11**	7–24	1.27
Jams and conserves	**14**	7–41	**14**	7–28	**19**	7–41	**11**	7–21	**14**	7–28	**14**	7–14	**14**	7–24	**12**	7–15	1.00
Chocolate	**25**	18–50	**30**	16–53	**36**	20–55	**26**	15–50	**25**	13–50	**25**	13–50	**20**	13–51	**16**	10–30	1.12
Sugar, white	**8**	4–13	**7**	4–10	**7**	4–13	**6**	4–8	**7**	4–9	**5**	4–8	**6**	4–8	**5**	4–8	1.17
Potato crisps	**42**	15–75	**35**	17–46	**23**	19–50	**21**	14–45	**21**	19–46	**20**	14–45	**19**	5–100	**15**	8–35	1.33
Potatoes, fries/wedges	**99**	57–128	**95**	57–137	**85**	57–128	**70**	31–104	**64**	31–115	**71**	29–110	**57**	29–72	**69**	29–85	1.03
Mayonnaise	**14**	14–21	**14**	14–21	**20**	14–21	**20**	14–21	**17**	14–21	**20**	14–21	**14**	8–19	**14**	14–20	1.00
Tomato sauce	**21**	14–42	**14**	14–21	**14**	14–28	**14**	11–21	**14**	14–21	**14**	14–37	**14**	12–14	**16**	14–44	1.00
Hamburgers	**238**	206–311	**196**	155–304	**256**	195–350	**207**	155–327	**301**	214–350	**214**	155–317	**186**	146–228	**175**	99–280	1.25
Pizza	**281**	185–375	**188**	100–281	**300**	162–400	**185**	100–281	**299**	162–375	**185**	98–325	**195**	153–238	**153**	91–344	1.60
Savoury pastries	**175**	130–175	**175**	126–351	**175**	130–175	**140**	74–175	**175**	130–192	**158**	81–175	**175**	130–178	**135**	42–175	1.09
Beverages																	
Beers, regular alcohol	**1000**	379–1419	**379**	303–556	**758**	379–1215	**379**	333–822	**758**	379–1152	**379**	344–758	**429**	379–761	**388**	368–758	2.00
Cordials	**473**	368–735	**353**	263–473	**464**	341–936	**368**	305–591	**368**	262–756	**312**	252–378	**361**	210–390	**263**	206–267	1.29
Fruit drinks	**364**	260–468	**302**	208–374	**302**	257–364	**286**	208–374	**302**	208–364	**260**	208–364	**260**	208–364	**237**	156–310	1.10
Soft drinks, regular	**390**	369–530	**390**	299–421	**390**	362–473	**364**	279–423	**390**	311–468	**364**	208–390	**302**	206–390	**267**	206–358	1.07
Wines, red/white	**297**	213–371	**312**	208–495	**347**	248–495	**297**	188–475	**361**	248–594	**297**	208–495	**248**	184–347	**240**	139–300	1.17

^1^All data were weighted to represent population estimates. IQR: Interquartile range; M: F Male to female ratio; sample sizes varies within age/sex group as median intake were based on per consumer. Significance of sex- and age- differences in typical portion sizes was tested by Mann-Whitney test and Kruskal-Wallis test respectively. All food categories had significant sex- and age-differences except for ham, tomato sauce, mayonnaise, jam and conserves.

**Table 3:** Comparison of median portion sizes (grams) for commonly consumed core foods among adults 19 years and over (2011–12 NNPAS) with Australian Dietary Guidelines (ADG) standard serves (grams and household measures)^1^.

	Male, 19 + years					Female 19 + years				
	n	NNPAS		ADG			NNPAS		ADG	Percent difference^*^
		Median	IQR	Standard serve	Percent difference^*^	n	Median	IQR	Standard serve	
Bread and cereals										
Bread rolls, white	372	69	69–69	40(1/2 medium)	73	324	69	58–69	40(1/2 medium)	73
Bread, white	1357	64	54–81	40(1 slice)	60	1399	54	38–66	40(1 slice)	35
Bread, wholemeal	627	66	56–84	40 (1 slice)	65	780	58	42–66	40(1 slice)	45
Breakfast cereal, all	1596	51	34–81	30(2/3 cup)	70	1716	35	27–60	30(2/3 cup)	17
Oats, cooked	262	218	156–402	120(1/2cup)	82	495	201	130–312	120(1/2cup)	68
Rice bubbles/Corn flakes	219	35	23–39	30(2/3 cup)	17	197	33	22–35	30(2/3 cup)	10
Rice, cooked	569	201	134–300	120(1/2cup)	68	633	137	84–207	120(1/2cup)	14
Wheat flake biscuits	599	51	34–68	30(2/3 cup)	70	447	34	26–40	30(2/3 cup)	13
Meat and alternatives										
Beef steak, cooked	492	170	142–200	65	162	445	150	104–180	65	131
Chicken, cuts, cooked	433	171	105–225	80	114	516	113	78–186	80	41
Eggs, whole	585	51	44–88	120(2 large)	−58	567	49	44–82	120(2 large)	−59
Fish, cooked	219	110	75–169	100	10	253	107	86–125	100	7
Lamb, cuts, cooked	227	127	78–161	65	95	244	104	72–152	65	60
Nuts	508	28	19–36	30	−7	651	27	12–36	30	−10
Milk and alternatives										
Cheese, cheddar, full fat	754	25	21–45	40 (2 slices)	−38	894	25	21–32	40 (2 slices)	−38
Milk, full fat, beverage	1319	31	31–62	258 (1cup)	−88	1299	31	72–206	258 (1cup)	−88
Milk, full fat, on cereal	661	193	100–258	258 (1cup)	−25	521	129	31–129	258 (1cup)	−50
Yoghurt, flavoured, full fat	140	154	92–200	200(3/4cup)	−23	215	123	66–175	200(3/4cup)	−39
Fruit										
Apples	816	164	154–180	150(1 medium)	9	995	164	143–164	150(1 medium)	9
Bananas	740	98	98–111	150(1 medium)	−35	1006	98	74–98	150(1 medium)	−38
Fruit juices	673	273	210–368	150(1/2 cup)	108	774	263	153–315	150(1/2 cup)	101
Grapes	208	88	50–170	150 (1 cup)	−41	349	66	39–136	150 (1 cup)	−56
Mandarins	253	75	75–150	150(2 small)	−50	344	75	75–113	150(2 small)	−50
Pears	213	181	176–200	150(1 medium)	21	276	181	153–181	150(1 medium)	21
Vegetables										
Broccoli, cooked	231	58	31–98	75(1/2 cup)	−23	375	41	93–95	75(1/2 cup)	−45
Carrot, cooked	377	60	30–106	75(1/2 cup)	−20	539	39	25–78	75(1/2 cup)	−48
Cucumber, raw	210	26	17–35	75(1 cup)	−65	280	25	17–38	75(1 cup)	−67
Green peas	258	40	26–80	75(1/2 cup)	−47	291	38	13–80	75(1/2 cup)	−49
Mixed non-leafy vegetables	436	143	72–185	75(1/2 cup)	91	508	114	71–171	75(1/2 cup)	52
Potatoes, boiled	506	172	104–234	75(1/2 medium)	129	605	112	83–203	75(1/2 medium)	49
Salad, leafy vegetables	648	78	50–130	75(1 cup)	4	971	65	46–115	75(1 cup)	−13
Tomato, raw	509	35	29–58	75(1 medium)	−53	524	29	29–38	75(1 medium)	−61
Unsaturated spreads and oils										
Margarine, monounsaturated	656	8	5–10	10(2tsp)	−20	730	7	5–10	10(2tsp)	−30
Margarine, phytosterols	176	5	5–10	10(2tsp)	−50	224	5	5–10	10(2tsp)	−50
Margarine, polyunsaturated	292	7	5–10	10(2tsp)	−30	327	5	5–10	10(2tsp)	−50
Margarine, total	1186	7	5–10	10(2tsp)	−30	1355	5	5–10	10(2tsp)	−50
Margarine, unspecified	107	5	5–10	10(2tsp)	−50	111	5	5–10	10(2tsp)	−50
Olive oil	132	18	9–32	7(1tsp)	157	165	18	9–18	7(1tsp)	157

^*^Percentage difference calculated as (NNPAS median serve – ADG standard serve)*100/ADG standard serve, P < 0.0001 (one sample Wilcoxon Signed Rank tests).

^1^All data were weighted to represent population estimates. IQR: Interquartile range, tsp: teaspoon.

**Table 4:** Comparison of median typical portion sizes and interquartile range (grams) for commonly consumed discretionary foods among adults 19 years and over (2011–12 NNPAS) with Australian Dietary Guidelines (ADG) 600 kJ standard serve for discretionary foods^1^.

	Male 19 + years						Female 19 + years					
	Portion size (g)			Energy per portion (kJ)		Percent Difference	Portion size (g)			Energy per portion (kJ)		Percent Difference
	n	Median	IQR	Median	IQR		n	Median	IQR	Median	IQR	
Tomato sauce	316	14	14–28	61	61–122	−90	237	14	14–21	61	61–90	−90
Ham	573	17	17–34	79	79–159	−87	558	17	17–25	79	79–134	−87
Sugar, white	1272	7	4–12	108	67–194	−82	1177	6	4–8	90	67–134	−85
Jams and conserves	378	14	7–28	154	77–307	−74	489	14	7–16	129	77–159	−79
Honey	363	14	7–29	188	94–377	−69	393	11	7–20	151	94–163	−75
Butter and dairy blends	760	7	5–10	218	145–291	−64	1002	5	5–10	146	145–290	−76
Savoury biscuits	521	20	12–34	348	202–628	−42	927	17	11–27	303	187–483	−50
Mayonnaise	310	20	14–21	390	334–576	−35	435	20	14–21	349	292–503	−42
Fruit drinks	345	312	208–392	542	387–732	−10	364	284	208–364	503	366–658	−16
Sweet biscuits	887	28	16–39	561	302–776	−7	1149	19	15–33	388	287–685	−35
Cordials	294	459	335–763	584	399–1043	−3	253	357	263–473	414	149–574	−31
Potato crisps	290	28	19–50	595	388–1080	−1	251	21	14–45	454	298–944	−24
Chocolate	636	28	16–50	602	331–997	0	886	25	15–50	545	320–978	−9
Soft drinks, sugar-sweetened	913	390	343–507	655	524–786	9	759	364	260–390	575	427–655	−4
Ice-cream	578	84	74–138	845	542–1149	41	571	79	64–102	651	440–940	9
Potatoes, fries/wedges	579	74	57–128	933	540–1402	56	466	72	36–114	835	363–1187	39
Wines, red/white	641	347	238–495	1036	710–1603	73	914	297	188–475	876	590–1450	46
Beers, regular alcohol	806	758	279–1212	1083	541–1733	81	156	379	333–756	542	503–1061	−10
Cakes, buns, muffins	600	110	65–163	1532	882–2190	155	830	95	49–142	1282	747–2042	114
Savoury pastries	426	175	130–175	1592	1414–1720	165	333	160	80–175	1592	793–1701	165
Sausages and frankfurts	352	152	89–200	1629	913–2072	172	261	101	89–178	1082	913–1993	80
Hamburgers	345	254	197–345	2425	2070–2838	304	257	203	155–306	2025	1627–2715	238
Pizza	212	291	164–375	3103	1872–4106	417	254	188	100–281	2005	1095–2987	234

*Percentage difference calculated as (NNPAS energy per portion–600 kJ)*100/600kJ, P < 0.0001 (one sample Wilcoxon Signed Rank tests).

^1^All data were weighted to represent population estimates. IQR: Interquartile range.
